# Diagnose and treatment of traumatic pleural-subarachnoid fistula in children: A case report and systematic review

**DOI:** 10.1186/s41016-020-00204-2

**Published:** 2020-08-06

**Authors:** Wei Yang, Ming Ge, Chenghao Chen, Qi Zeng

**Affiliations:** 1 Department of Neurosurgery, Beijing Children’s Hospital, Capital Medical University, National Center for Children’s Health, Beijing, 100045 China; 2 Department of Thoracic, Beijing Children’s Hospital, Capital Medical University, 56 South Lishi Road, Xicheng District, Beijing, 100045 China

**Keywords:** Pleural-subarachnoid fistula, Intracranial hypertension, Treatment, Complications

## Abstract

**Background:**

Pleural-subarachnoid fistula (PSF) is a rare disease that is difficult to diagnose and treat. Secondary intracranial hypertension and the treatment are seldom mentioned previously among PSF.

**Case presentation:**

A 1-year-old boy diagnosed PSF developed into secondary intracranial hypertension after conservative treatment. He was finally cured by down-step treatment of mannitol, avoiding form ventricle-peritoneal shunt. Then, we reviewed the literature of pleural-subarachnoid fistula. Fifty-six cases have been reported so far. Most of the cases (51.8%) were caused by surgery; only 17.9% were caused by car accidents. Regarding the treatment, half of the cases cured by surgery and the other by conservative measures. Our case is the first one involving secondary intracranial hypertension and cured by down-step treatment of mannitol.

**Conclusions:**

A comprehensive examination should be performed before the treatment to avoid any inappropriate medical strategies. Secondary acute intracranial hypertension may be cured by down step treatment of mannitol, evading from the long-term ventriculoperitoneal shunt.

## Background

Pleural-subarachnoid fistula is a rare and complicated disease, which can be iatrogenic, traumatic, and spontaneous. The symptoms and signs could be a postural headache, mental changes, and dyspnea. The imaging examinations also present variously. Not all imaging examinations of pleural-subarachnoid fistula (PSF) showed pleural effusion; some only showed no significant changes in the pleural cavity or dilatation of the upper mediastinum [1, 2]. A small number of patients had mental changes. The imaging examination will show the pneumocranium on the CT scan and the pneumothorax on the chest X-ray [3–10]. Myelography, CT myelography, spinal MR, β-2 transferrin, and biochemical analysis of pleural effusion can be used to assist the diagnosis of PSF. As for treatment, surgery and non-surgery treatment both have been reported as an available measurement. In this article, we described a 1-year-old boy with persistent pleural effusion caused by a car accident who was confirmed to be PSF by myelography. PSF was cured by the lumbar cistern and thoracic drainage for about 2 months. After that, he experienced secondary acute intracranial hypertension. A down step of mannitol was used, and the intracranial hypertension was under control finally. After a 1-year follow-up, there are no any recurrent of PSF and intracranial hypertension signs.

## Case presentation

### Medical history

A 21-month-old boy with thorax drainage was admitted to our medical center for massive pleural effusion lasting for 1 month approximately for an unknown reason. One month ago, the boy experienced a car accident, and his chest hit against the airbag. Then, he was sent to the local emergent rescue. An instant CT scan showed a hydrops area in the abdominal and chest. Then, an exploratory laparotomy was performed, and spleen rupture and retroperitoneal hematoma were found during the surgery. The boy experienced a splenectomy, and drainage was placed for pleural in the thorax. Subsequently, he experienced persistent pleural effusion. The chest tube drained 400–600 ml every day, and the fluid was clear. The child kept a good mental state and did not complain about anything. He had been fasting for a few days in the previous hospital for the consideration of chylothorax diagnosis, but the pleural effusion did not reduce obviously. In our medical center, chylothorax, pleural-subarachnoid fistula, and urine leakage were taken into differential diagnoses according to the history of trauma and surgery.

The biochemical analyses of the thorax fluid are as follows: Rivalta test (−); WBC, 64 × 10^6^/L; mononuclear cells counts, 14; multinucleated cell counts, 50; total protein, 1.5 g/L; lactic dehydrogenase, 61 U/L; adenosine deaminase, 0.1 U/L; glucose, 4.64 mmol/L; and creatinine, 33.3 umol/L.

**Fig. 1:**
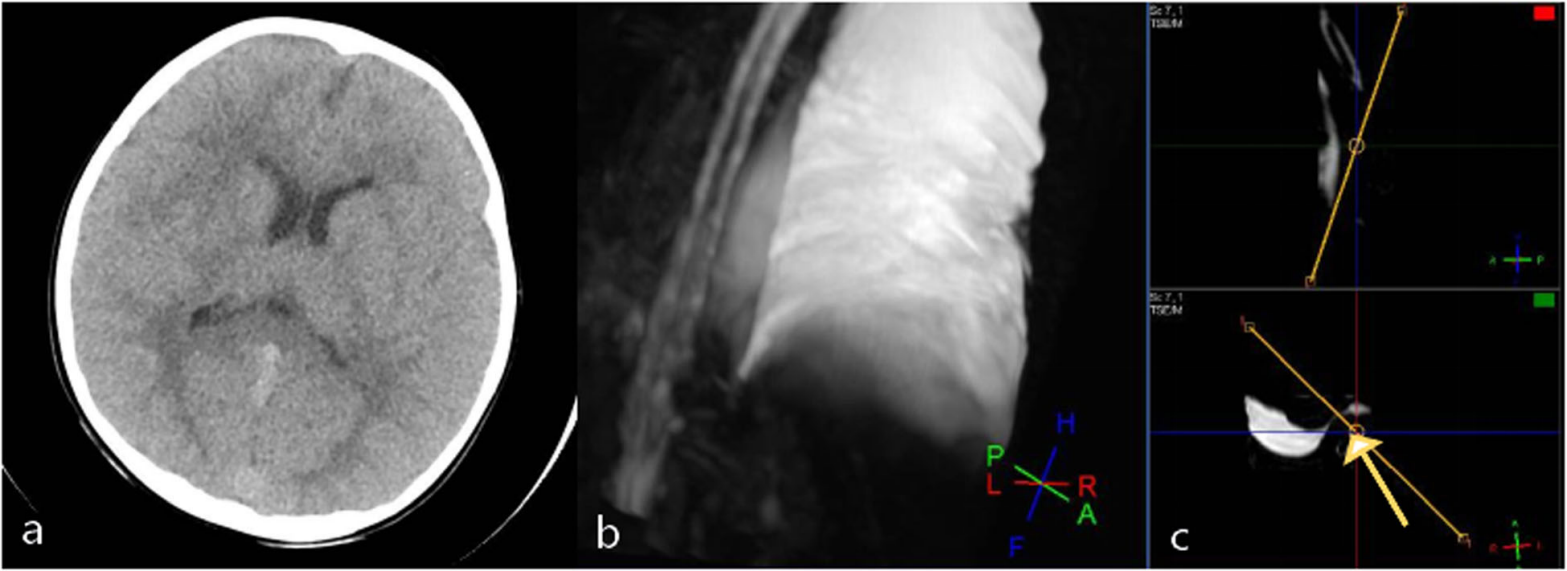
**a**–**c** Brain CT and heavily T2 spine MR. **a** No abnormal signs on brain CT. **b**, **c** The arrow in heavily spine MR shows the suspected fistula signal between at T11 level

The chest radiography showed massive pleural effusion in the right thorax. B ultrasound of the urinary system did not show any abnormity. The heavily T2 MR of the spine suspected a fistula at the T11 level. Brain CT did not present any positive findings (Fig. 1).

### Treatment and follow-up result

**Fig. 2:**
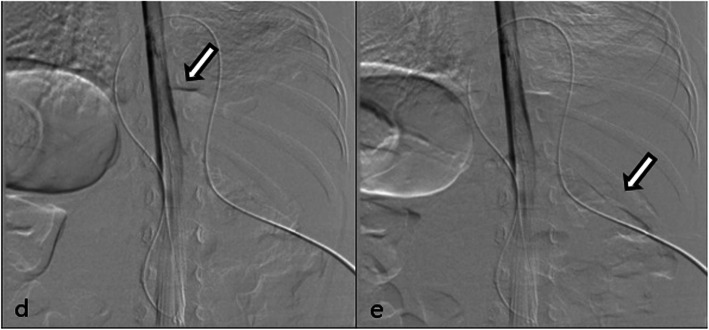
**d**–**e** Myelography. **d**–**e** The arrows show the contrast agents leaking from the subarachnoid space to the thorax

**Fig. 3:**
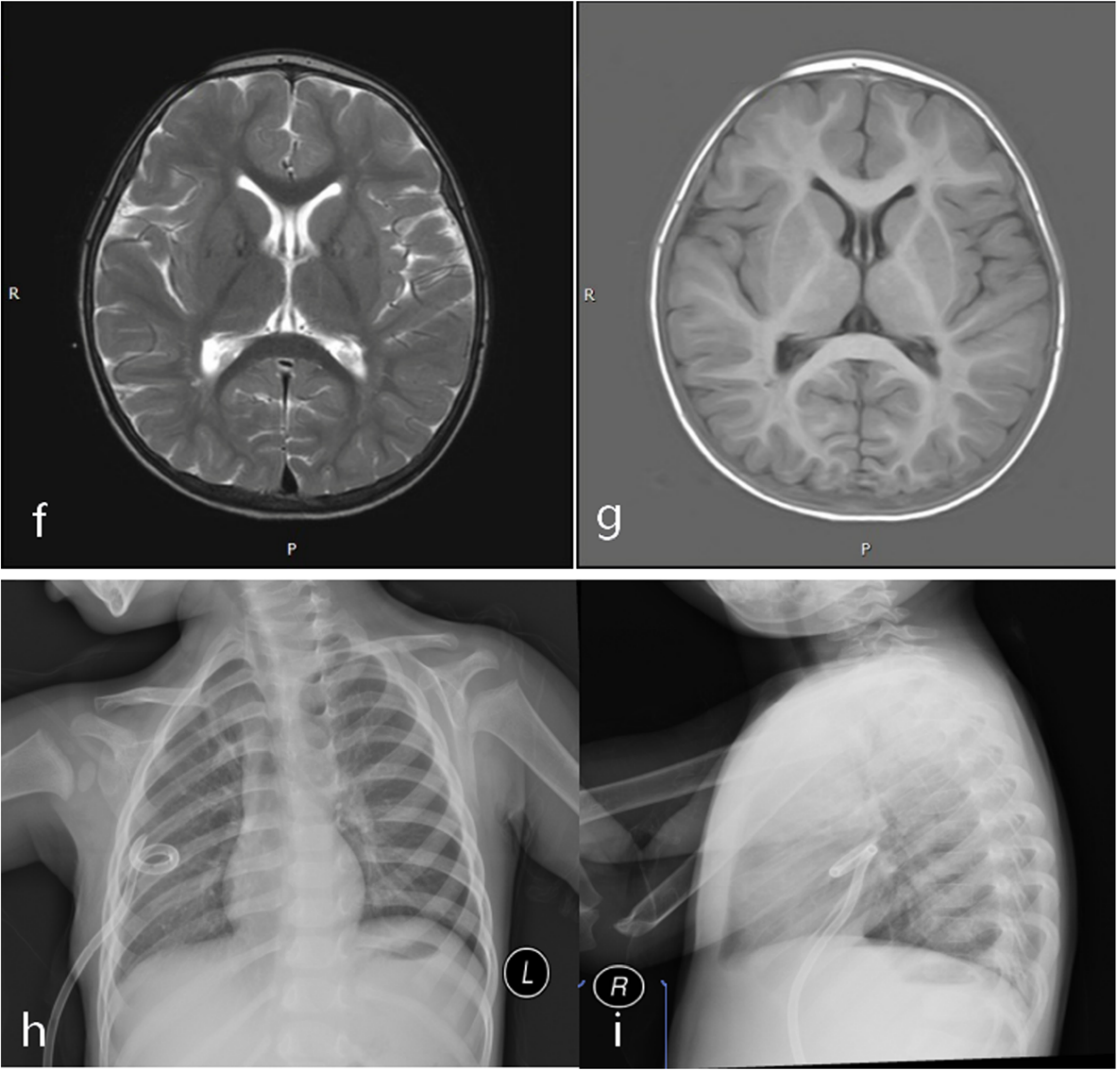
**f**–**i** Chest X-ray and brain MRI. **f, g** The MRIs show no ventriculomegaly or paraventricular edema after removal of cisternal drainage. **h**, i The chest X-ray show the no effusion in the thorax

To distinguish it from chylothorax, the child was allowed to eat some food. However, we did not see any changes to the drainage. Combined with the negative Rivalta test and clear color of drainage, the chylothorax was excluded. Furthermore, the creatinine was not at a similarly high level as the urine. Due to a positive finding from heavily T2 spine MR, the most likely diagnosis would be PSF. A thorax endoscopy exploratory surgery was performed to demonstrate the diagnosis and expecting to find the fistula and repair it. However, we failed to repair the fistula due to the desperate difficulty to find the lesion. To further confirm the diagnosis of PSF, myelography was performed and reassured the pleural-subarachnoid fistula at T11 level (Fig. 2). Meanwhile, we performed lumbar cisternal drainage to relieve the pressure of the cerebrospinal fluid (CSF), which could benefit the natural repairment of PSF theoretically. The drainage of the thorax and lumbar cisternal were about 200 ml and 460 every day. The total volume always went around 400–600 ml. Three weeks later, the drainage of the thorax decreased to nearly zero suddenly, but the drainage of the lumbar cisternal was around 400–600 ml. Two more weeks later, we removed the chest tube and elevated the level of the lumbar cisternal drainage gradually, and there was no pleural effusion ever (Fig. 3). The drainage volume decreased as the height elevated. We removed the lumbar cisternal drainage 5 days later. During this period, the antibiotic was used to prevent infection, so the child did not catch any infection.

One day after the removal of the lumbar cisternal drainage, the boy became agitated and fell into lethargy. Acute intracranial hypertension was considered due to the lumbar puncture showing more than 300 mm H_2_O of pressure, and the test of CFS did not show the features of infection. So mannitol was given 10 ml/kg Q8h. An MR scan of the head did not show dilation of the ventricle and paraventricular edema (Fig. 3). The boy got back to normal state quickly, and his mental status got worse when we stopped the mannitol treatment at the early stage. This meant that he was involved in stubborn intracranial hypertension and mannitol had become a necessity for him. A ventriculoperitoneal shunt once was considered as a treatment to the secondary acute intracranial hypertension. However, his parents refused to carry the movement. Then, we tried the down step of mannitol treatment, and we stopped the mannitol 2 weeks later. The boy did not complain about any headache. And the re-chest X-ray was normal. After 1 year following, he has not shown pleural effusion again and intracranial hypertension.

A total of 56 cases were initially retrieved through the database search. All the papers were fully read, and we collected and summarized all the etiology, manifestations, detection measures, and treatment methods. The most common cause of PSF (Table 1) was medical operation (51.8%), especially in spinal operation. The following causes were injury (26.8, especially refer gunshot), car accident (17.9%), and other rare events (3.6%, such as massage, tumor infiltration). As for clinical manifestation (Table 2), continuous or massive pleural effusion was the most common one (74.1%). About 10.3% of patients show pneumocephalus, and 8.6% of patients present with hemopneumothorax. Only a few patients featured by posture headache or upper mediastinum. There were some techniques (Table 3) used by doctors to make a diagnosis of PSF. Radioisotope myelography is the most effective means. Heavy T2-weighted myelography MR is a new non-invasive method to detect the fistula with a positive rate at 88.9%. The other techniques are β2 transferrin (85.7%), CT myelography (83.3%), and myelography (66.7%). For all the 56 patients, 57.1% of the patients received surgical treatment (Table 4), including through the thorax route (40.8%) and through the laminar route (14.3%). The other patients were cured by different kinds of conservative treatments, and the most popular one was drainage.

**Table 1:** Etiology of PSF

Etiology	Percentage (number)
Iatrogenic	51.8% (29)
Injury	26.8% (15)
Car accident	17.8% (10)
others	3.6% (2)

**Table 2:** Manifestation of PSF

Manifestation	Percentage (%)
Pleural effusion	74.1
Pneumocephalus	10.3
Hemopneumothorax	8.6
Postured headache	5.3
Upper mediastinum dilation	1.7

**Table 3:** Examination technique for diagnosis

Examination technique	Positive rate (%)
Radioisotope myelography	100
Heavy T2-weighted myelography MR	88.9
β2 transferrin	85.7
CT myelography	83.3
Myelography	66.7

**Table 4:** Treatment of PSF

Treatment	Percentage (%)
Surgery	57.1
Through thorax	40.8
Through laminar	14.3
Thorax-peritoneal shunt	2.0
Non-surgery	42.9
Drainage	24.5
NPPV	10.2
Injection of the biological glue	4.1
Epidural blood patch	4.1

## Discussion

The pleural-subarachnoid fistula was first reported by Milloy et al. [11] in 1959. The clinical manifestations of PSF can be very diverse. Continuous pleural effusion (74.1% of the cases) is the most common manifestations of PSF. Pneumocephalus and pneumothorax without pleural effusion consist of 10.3% of the cases [8]. They remain present with hemopneumothorax postured headache [12], or just a dilation of the upper mediastinum [2]. The cerebrospinal fluid is sucked into the thorax due to the lower pressure in the thorax cavity. Besides, pleural effusion may come from the thoracic lymphatic system, urine from the injured urinary system, transudate from the vascular system, infectious exudate. PSF, urine, and chylothorax are always characterized by continuous liquid or massive volume. The difference is the color of the liquid. PSF is generally clear, and urine can be clear or urine color, and chylothorax is always milky and will change with food intake. Apart from the color, a negative Rivalta test could be used to distinguish transudate and exudate. PSF thus should be taken into consideration in continuous pleural effusion circumstance, especially when the Rivalta test is negative. However, to confirm the diagnosis, further examinations should be taken.

Several techniques have been developed to assist the diagnosis of PSF, which include β2 transferrin, special MR of the spin, myelography, CT myelography, and radioisotope myelography. In this review, we made a summary of the sensitivity of all the techniques and found that they should be performed in an appropriate sequence. In terms of positive rate, radioisotope myelography (100%) is the most effective method. In this review, all the patients who did this examination showed positive results. However, this kind of an examination has more radiation damage to the human body, compared with other measures. There is a special sequence of MR which has been applied to discover the PSF, with a positive rate at 88.9%, higher than CT myelography and myelography, which are at 83.3% and 66.7%, respectively. Nearly every technique has a potential negative rate, so they should be used with a comprehensive sequence during the diagnose procedure according to sensitivity and harmfulness. β2 transferrin should be taken into consideration firstly as a non-invasive and handy test. If these examinations fail, and a PSF is strongly suspected, the probe surgery can be performed for diagnosis and treatment. However, fistulas were not visible during surgery [3, 13] sometimes.

Treatments for PSF include surgery and non-surgery. Although surgical treatment is a little more common than non-surgery strategy, there are more kinds of conservative measurements including thorax drainage, lumbar cisterna drainage, keeping supine position [10], epidural blood patch [12, 14], positive pressure ventilation [15–18], biological glue injection percutaneously [19, 20], and so on. Percutaneous injection of biological glue was newly reported treatment. As a minimally invasive treatment, it will be a promising strategy. In a word, doctors are attempting to find more and more conservative methods to treat the PSF due to the difficulty to find the lesion during operation, especially for traumatic PSF. Of course, open surgery is suitable for large fistula, which is difficult to be cured by non-surgical measures [21]. Each method has its pros and cons. Doctors should choose a suitable method according to medical history and the type of fistula. Of course, Conservative strategies may spend more hospital days and have a risk of infection.

Complications related to the treating measure were seldom reported before. Knafo et al. [20] reported the thorax pain after injection of onyx. Infection [22] and neurological defects [4] were reported separately by Wilson and Jumer and Brown and Symbas. Milloy et al. [11] reported a 19-year-old man complaining headache after repairing the pleural-subarachnoid fistula. The reason may be that this patient experienced pleural effusion for about 8 months. The balance of cerebral-spinal fluid (CSF) circulation had changed before surgery, so the closure of the fistula might result in intracranial hypertension. In our case, the patient progressed headache and coma after the removal of the lumbar cisternal drainage. A lumbar puncture was carried, and it demonstrates the patient was in hyper intracranial tension state. This may be because long-term of cisternal drainage has created a new balance of CSF. After removing the cisternal tube, CSF could not outflow from the lumbar cisternal tube, so CSF pressure increased sharply. And this caused a headache and coma state. Generally, doctors would perform V-P shunt as a solution to this situation. We once managed to do so. But the patient’s parents refuse to do so. So we tried the down step of mannitol treatment; the boy was cured finally. In fact, among the published article, researchers always perform the V-P shunt in the similar situations. A down step of mannitol treatment is seldom described to treat such serious secondary acute intracranial hypertension. This indicates that children have a more powerful ability to build a new balance of CSF. And in the face of secondary intracranial hypertension, we should choose V-P shunt more carefully.

## Conclusion

The PSF is a very rare situation that can be difficult to diagnose and treat. PSF should be taken into consideration in a continuous pleural effusion situation. A full-scale and sequenced examination should be performed to assist the diagnosis of PSF. The treatment strategy should be based on clinical features. Intracranial hypertension after the closure of the fistula should be treated seriously. The down-step of mannitol should be tried first before the V-P shunt.

## Data Availability

Please contact the author for data request.

## Abbreviations

PSF: Pleural subarachnoid fistula
NPPV: Noninvasive positive pressure ventilation
CSF: Cerebrospinal fluid
CT: Computer tomography
MR: Magnetic resonance
